# Development and Validation of a Stability-Indicating High Performance Liquid Chromatographic (HPLC) Method for the Determination of Related Substances of Micafungin Sodium in Drug Substances

**DOI:** 10.3390/ijms141121202

**Published:** 2013-10-24

**Authors:** Shengsheng Zhu, Xiang Meng, Xin Su, Yongwei Luo, Zuyue Sun

**Affiliations:** 1 School of Pharmacy, Fudan University. Shanghai 200032, China; E-Mails: zhushengsheng99@126.com (S.Z.); 11111150005@fudan.edu.cn (X.M.); suxin198383@163.com (X.S.); luoyongwei88@163.com (Y.L.); 2 Department of Pharmacology and Toxicology, Shanghai Institute of Planned Parenthood Research, Shanghai 200032, China

**Keywords:** micafungin sodium, related substances, stability-indicating, forced degradation, validation

## Abstract

An isocratic, sensitive and stability-indicating high performance liquid chromatographic (HPLC) method for separation and determination of the related substances of micafungin sodium was developed. The chromatographic separation was achieved on Agilent Zorbax SB-C18 column (250 × 4.6 mm, 5 μm). Forced degradation study confirmed that the newly developed method was specific and selective to the degradation products. The performance of the method was validated according to the present ICH guidelines for specificity, linearity, accuracy, precision and robustness. Regression analysis showed correlation coefficient value greater than 0.999 for micafungin sodium and its six impurities. Limit of detection of impurities was in the range of 0.006%–0.013% indicating the high sensitivity of the newly developed method. Accuracy of the method was established based on the recovery obtained between 98.2% and 102.0% for all impurities. RSD obtained for the repeatability and intermediate precision experiments, was less than 1.0%. The method was successfully applied to quantify related substances of micafungin sodium in bulk drugs.

## 1. Introduction

**Figure 1:**
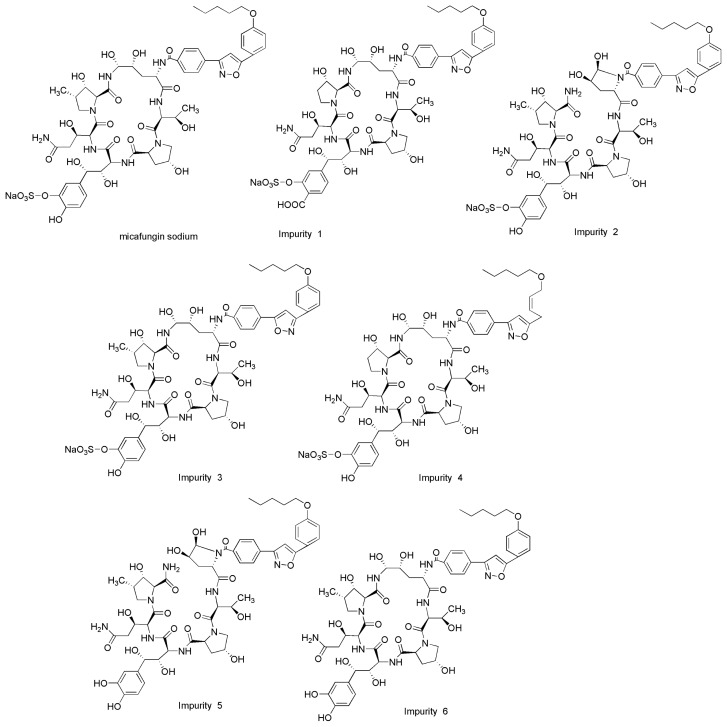
Chemical structures of micafungin sodium and its impurities 1–6.

Micafungin sodium is a cyclic semisynthetic derivative of the echinocandin-like lipopeptide FR-901379 isolated from the culture broth of *Coleophoma empetri*, a plant pathogen associated with postharvest fruit rot in cranberries [1,2] It has an empirical formula of C_56_H_70_N_9_NaO_23_S, a molecular weight of 1292.26 g/mol [3]. The chemical structures of micafungin sodium and its related substances (namely imp-1, imp-2, imp-3, imp-4, imp-5 and imp-6) are presented in Figure 1. Micafungin sodium has been approved for the treatment of esophageal candidiasis, and for the prophylaxis of Candida infections in patients undergoing hematopoietic stem cell transplantation [4,5]. Micafungin sodium has a unique mechanism of action that inhibits the synthesis of 1,3-β-d-glucans in the fungal cell wall [5–7]. The drug was first launched in Japan in December 2002 as Fungard™ [8]. It was then also approved as Mycamine™ by the US Food and Drug Administration in March 2005 [9].

The presence of impurities in active pharmaceutical ingredients for drugs can have a significant impact on the quality and safety of the drug products. Therefore, it is necessary to study the impurity profiles of drug substances to be used in the manufacturing process of drug products [10,11]. A few HPLC methods have appeared in the literature for the quantification of micafungin in plasma [12–16]. Several other methods have been published for the quantfication of two active metabolites of micafungin simultaneously [17,18]. To the best of our knowledge, there is no stability-indicating HPLC method reported in the literature that can conduct an accurate and quantifiable analysis of degradation products and related substances of micafungin sodium. It is, therefore, necessary to develop a new stability-indicating method for the determination and quantitative estimation of related substances of micafungin sodium.

Hence, a reproducible stability-indicating HPLC method was developed for the quantitative determination of related substances of micafungin sodium. This method was successfully validated with respect to specificity, Limit of detection (LOD), limit of quantification (LOQ), linearity, precision, accuracy and robustness. Forced degradation studies were performed on the drug substance to show the stability-indicating nature of the method. These studies were performed in accordance with the ICH guidelines [19,20].

## 2. Results and Discussions

### 2.1. Method Development

**Figure 2:**
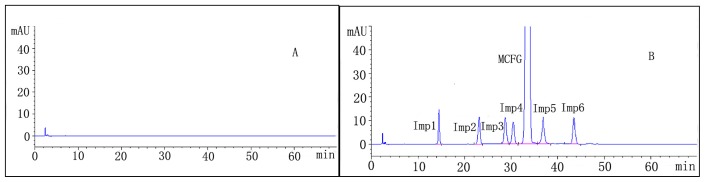
Representative chromatograms of (**A**) diluent, (**B**) micafungin sodium spiked with 0.5% of impurities 1–6.

To develop a rugged and suitable HPLC method for the quantitative determination of micafungin sodium and its related substances, the analytical conditions were selected after testing different parameters such as diluents, buffer, buffer concentration, organic solvent for mobile phase, mobile phase composition and other chromatographic conditions. Our preliminary trials, using different compositions of mobile phases consisting of water with methanol or acetonitrile, did not give good peak shapes. By using 0.01 M sodium dihydrogen phosphate and 0.05 M sodium perchlorate buffer, adjusted to pH 2.9 with phosphoric acid and keeping the mobile phase composition as acetonitrile-buffer (38:62, *v*/*v*), the best peak shape was obtained. For the selection of organic constituent of the mobile phase, acetonitrile was chosen to attain good peak shapes. Typical chromatograms are presented in Figure 2.

### 2.2. Method Validation

The validation of the optimized method was performed in agreement with the ICH guidelines [20]. The following parameters were considered: specificity, linearity, accuracy, precision, LOD and LOQ, and robustness. A system suitability test was used to evaluate routine method performance.

#### 2.2.1. System Suitability

The evaluation of the method ability to produce good resolution between the peaks of interest with high repeatability was determined by injecting five replicate of freshly prepared micafungin sodium spiked with 0.5% of impurities 1–6. The chromatogram was analyzed regarding its resolution (*R*), theoretical plates (*N*), symmetry factor and retention time (*t*_R_). The results of system suitability test show that the proposed method fulfils the requirements within the accepted limits (Table 1).

#### 2.2.2. Specificity

Specificity of a method can be defined as absence of any interference at retention times of peak of interest, and was evaluated by observing the chromatograms of blank samples and samples spiked with micafungin sodium and impurities 1–6. The chromatogram of micafungin sodium spiked with impurities 1–6 shows no interference of impurities with drug substance. Suitability parameters and resolution values were presented in Table 1. The chromatogram of micafungin sodium spiked with impurities 1–6 for specificity is presented in Figure 2.

#### 2.2.3. Precision

The precision of the related substance method was checked by injecting six individual preparations of micafungin sodium (1000 μg/mL) spiked with 0.50% each of imp-1, imp-2, imp-3, imp-4, imp-5 and imp-6 with respect to the micafungin sodium analyte concentration. RSD (%) of peak area was calculated for the impurities. The intermediate precision of the method was verified by a different analyst, on a different day, using an instrument of a different make, in the same laboratory. The precision datas were summarized in Table 2. From Table 2, it can be clearly seen that the intraday and interday % RSD of chromatographic determination were observed in the range of 0.16%–0.34% and 0.28%–0.55%, respectively. The low RSD values of repeatability and intermediate precision studies indicate that the method is precise for the determination of its related substances of micafungin sodium.

#### 2.2.4. Limit of Detection and Limit of Quantification

The LOQ and LOD detection of impurities 1–6 and micafungin sodium were determined by series of dilutions of stock solutions of each impurity and drug substance to attain an average signal-to-noise ratio of 3:1 and 10:1, respectively. Details of the detection and quantification limits of each impurity and drug substance are presented in Table 3. As shown in Table 3, the precision at the LOQ concentration for imp-1, imp-2, imp-3, imp-4, imp-5 and imp-6 was below 2%. For all impurities, the LOQ is <0.05%. It is shown that the method is sensitive and precise at very low concentrations of the analyte and its impurities.

#### 2.2.5. Linearity and Range

Linearity test solutions were prepared from impurities stock solution at seven different concentration levels ranging from LOQ, 25% (1.25 μg/mL), 50% (2.50 μg/mL), 75% (3.75 μg/mL), 100% (5.00 μg/mL), 125% (6.25 μg/mL) and 150% (7.50 μg/mL) of work concentration (5.0 μg/mL). Calibration curves were constructed by plotting the peak area against the concentration using linear regression analysis. Table 3 represents calibration characteristics for micafungin sodium and its impurities 1–6. From Table 3, it can be clearly seen that the correlation coefficient obtained was greater than 0.999. The confidence interval for intercept and residual standard deviation were also presented in Table 3. The results revealed an excellent correlation between the peak area and analyte concentration.

#### 2.2.6. Accuracy

Accuracy expresses the closeness of the agreement between the true value and the value obtained. The accuracy of the related substance method for the quantification of all six impurities (imp-1, imp-2, imp-3, imp-4, imp-5 and imp-6) in the bulk drug. The study was carried out in triplicate at 0.25%, 0.50% and 0.75% of the analyte concentration (1000 μg/mL). The percentage recovery of imp-1, imp-2, imp-3, imp-4, imp-5 and imp-6 in bulk drug samples ranged from 98.4% to 102.0%. The results are given in Table 4.

#### 2.2.7. Robustness

To determine the robustness of the developed method, experimental conditions were deliberately altered and the resolutions of micafungin sodium and all the impurities 1–6 were evaluated. The flow rate of the mobile phase was changed from 1.0 mL/min to 0.9 mL/min and 1.1 mL/min. The affect of column temperature on resolution was studied at 40 °C and 50 °C instead of 45 °C. The composition of organic solvent was changed from 38% to 37% and 39%. The affect of pH on resolution of impurities was also studied by varying ±0.2 pH units (at 2.7 and 3.1 buffer pH instead of 2.9). When the chromatographic conditions (flow rate, column temperature, the composition of organic solvent and pH) were deliberately varied, the resolutions of micafungin sodium and the impurities 1–6 were not significantly affected, illustrating the good robustness of the method. The results were given in Table 5.

#### 2.2.8. Solution Stability and Mobile Phase Stability

The solution stability and the mobile phase stability of micafungin sodium were tested up to 48 h period, by assaying the freshly prepared sample solutions against freshly prepared reference standard solutions for every 6 h interval up to two days. Mobile phase prepared was kept constant during the study period. The % RSD of the content of the impurities were calculated and was less than 1.0%. The solution stability and mobile phase stability experiments data confirms that sample solution and mobile phase used is stable up to 48 h.

### 2.3. Forced Degradation Behavior

**Figure 3:**
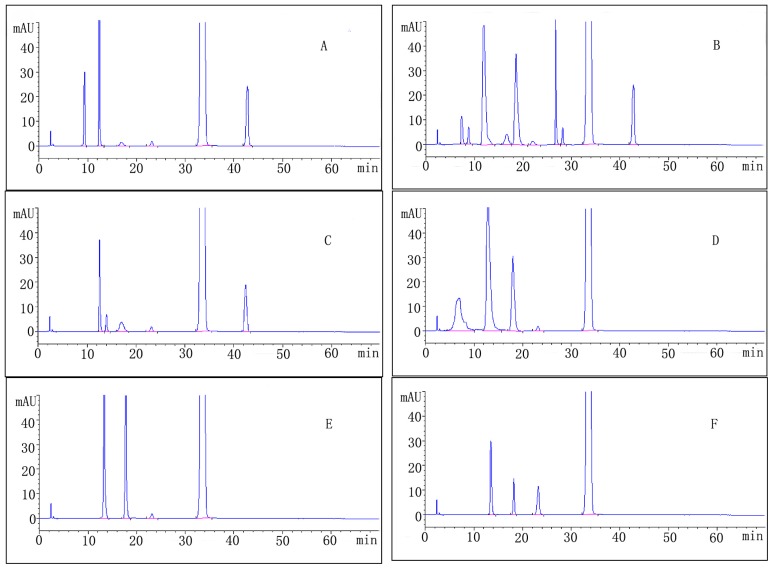
Chromatogram of micafungin sodium under stress conditions: (**A**) acid hydrolysis; (**B**) base hydrolysis; (**C**) aqueous hydrolysis; (**D**) oxidative degradation; (**E**) photolytic degradation and (**F**) thermal degradation.

The result of forced degradation studies of micafungin sodium with approximate percentage degradation and retention time of major degradation products is given in Table 6. Chromatograms of forced degradation study have been depicted in Figure 3.

#### 2.3.1. Acid Degradation

The drug was found to be moderately unstable to acid degradation. The known impurities in the study were found to be impurity 2 (0.2%) and impurity 6 (2.3%), with 6.1% of a maximum unknown degradant at a relative retention time (RRT) of approximately 0.36, with total impurities of approximately 11.1% (Table 6, Figure 3A).

#### 2.3.2. Base Degradation

The drug was found to be quite unstable to base degradation. The known impurities in the study were found to be impurity 2 (0.2%), impurity 3 (0.4%) and impurity 6 (2.1%), with a maximum unknown degradant (13.3%) at an RRT of approximately 0.36, with total impurities of approximately 25.1% (Table 6, Figure 3B).

#### 2.3.3. Water Degradation

The drug was found to be slightly unstable to water degradation. The known impurities in the study were found to be impurity 1 (0.8%), impurity 2 (0.2%) and impurity 6 (1.2%), with a maximum unknown degradant (2.2%) at an RRT of approximately 0.36, with total impurities of approximately 5.5% (Table 6, Figure 3C).

#### 2.3.4. Oxidation Degradation

The drug was found to be quite unstable to oxidation degradation. The known impurities in the study were found to be impurity 2 (0.2%), with a maximum unknown degradant (12.2%) at an RRT of approximately 0.36, with total impurities of approximately 17.6% (Table 6, Figure 3D).

#### 2.3.5. Photolytic Degradation

The drug was found to be moderately unstable under to photolytic degradation. The known impurities in the study were found to be impurity 2 (0.2%), with a maximum unknown degradant (4.5%) at an RRT of approximately 0.41, with total impurities of approximately 9.2% (Table 6, Figure 3E).

#### 2.3.6. Thermal Degradation

The drug was found to be slightly unstable to thermal degradation. The known impurities in the study were found to be impurity 2 (1.2%), with a maximum unknown degradant (2.8%) at an RRT of approximately 0.41, with total impurities of approximately 5.2% (Table 6, Figure 3F).

### 2.4. Application of the Method: Analysis of Bulk Drug

The validated method was applied to quantify impurities in three batches of micafungin sodium bulk drug (lots: 1228065, 1228066, 1228067), there was no interference from impurities with analysis of micafungin sodium. The results were listed in Table 7. It was found that the content of individual impurity is below 0.20% and that the total sum of impurities was below 0.40%. The reporting threshold was 0.05%.

## 3. Experimental

### 3.1. Instrumentation

The photodegradation was carried out in a photostability chamber (KBF 240, Binder, Tuttlingen, Germany) capable of controlling the temperature and humidity within a range of ±2 °C and ±5% RH, respectively. The chamber was equipped with illumination bank made of light source as described in option 2 in the ICH guideline Q1B [21]. The chamber was set at a temperature of 25 °C and humidity of 55%. Thermal stability studies were carried out in a dry air oven (Lindberg-Blue, Asheville, NC, USA).

Chromatographic analysis were performed on an Agilent HP1100 system (Agilent, Santa Clara, CA, USA), which consisted of a G1311A Quat pump, a G1322A vacuum degasser, G1313A Autosampler, G1315A diode array detector and a G1316A thermostatted column compartment. Chromatographic data were processed using the ChemStation software (Agilent, Santa Clara, CA, USA). Other apparatus included a DL-60D ultrasonic device (Shanghai, China), a PHS-3CW digital pH meter (Shanghai LIDA Instrument Factory, Shanghai, China) and a Milli-Q water purification system (Millipore, Bedford, MA, USA).

### 3.2. Materials and Reagents

Micafungin sodium was purchased from Aasenbo Pharm-Tech Co. Ltd. (Beijing, China). Related substances of micafungin sodium impurities 1–6 were provided by ShangPharma Co. Ltd. (Shanghai, China). Acetonitrile (HPLC grade) was purchased from Merck (Shanghai, China). Sodium hydroxide, hydrochloric acid, hydrogen peroxide solution 30% (*w*/*w*) in water and phosphoric acid (85%) were obtained from Sinopharm Medicine Chemical Reagent Co. Ltd. (Shanghai, China). Sodium dihydrogenphosphate (NaH_2_PO_4_), dibasic sodium phosphate (Na_2_HPO_4_) and potassium dihydrogenphosphate (KH_2_PO_4_) was provided by Sigma Aldrich Trading (Shanghai, China). Sodium perchlorate (NaClO_4_) was purchased from Aladdin Reagent Co. Ltd. (Shanghai, China). Water was purified using Milli-XQ equipment (Millipore, Bedford, MA, USA).

### 3.3. Chromatographic Conditions

The separation was carried out using an isocratic program on a C18 column (Agilent Zorbax SB, 250 × 4.6 mm, 5 μm, Agilent, Santa Clara, CA, USA). The mobile phase composition was pH 2.9 buffer (1.20 g of sodium dihydrogen phosphate and 6.15 g of sodium perchlorate in 1000 mL of water and adjusted to pH 2.9 with phosphoric acid) and acetonitrile in the ratio of 62:38 (*v*/*v*). The mobile phase was filtered through 0.45 μm membrane filter and degassed under ultrasonication. The flow rate was kept constant at 1 mL/min and the column was maintained at 45 °C. The detection was performed at 210 nm using a diode array detector and the injection volume was 10 μL. All calculations concerning the quantitative analysis were performed with external standardization by measurement of peak areas.

### 3.4. Preparation of the Solutions

#### 3.4.1. Diluent Solution

Diluent solution was prepared by mixing the phosphate buffer with acetonitrile HPLC grade, which is in the ratio of 1:1. The pH of the diluent solution was adjusted to the desired pH value (pH 6.5) by phosphoric acid. Phosphate buffer was prepared by mixing 2.72 g potassium dihydrogen phosphate with 2.84 g dibasic sodium phosphate in a 1000 mL beaker, the phosphate buffer was completed to mark by ultrapure water.

#### 3.4.2. Working Solution

Separate stock solutions of micafungin sodium and impurities (imp-1, imp-2, imp-3, imp-4, imp-5 and imp-6) at 1000 μg/mL were prepared in diluent solution. The stock solution was stored at 4 °C. The solutions were adequately diluted with diluent solution to study accuracy, precision, linearity, LOD and LOQ. Micafungin sodium working solution of 1000 μg/mL was prepared for the determinations of the related substances. All the solutions for analysis were prepared and analyzed freshly and all the samples were filtered through 0.22 μm membrane filter before analysis.

### 3.5. Forced Degradation Studies

Forced degradation studies were performed at a 1000 μg/mL concentration of micafungin sodium to provide an indication of the stability-indicating property and specificity of the proposed method. A peak purity test was conducted for the micafungin sodium peak by using a photodiode array detector on stress samples. All solutions used in forced degradation studies were prepared by dissolving the bulk drug in a small volume of stressing agents. After degradation, these solutions were diluted with diluent solution to yield a stated micafungin sodium concentration of approximately 1000 μg/mL. Conditions employed for performing the stress studies are described in the following.

#### 3.5.1. Acid Degradation

25.0 mg micafungin sodium were accurately weighed and dissolved in 5 mL of diluent, 5 mL 0.1 M HCl were added and the mixture was kept at room temperature for 1 h. Then the solution was neutralized by the addition of 5 mL 0.1 M NaOH and diluted to 25 mL with diluent.

To prepare the blank, 5 mL of 0.1 M HCl and 5 mL of 0.1 M NaOH were diluted to 25 mL with diluent.

#### 3.5.2. Base Degradation

Twenty-five milligram micafungin sodium were accurately weighed and dissolved in 5 mL of diluent, 5 mL 0.1 M NaOH were added and the mixture was kept at room temperature for 1 h. Then the solution were neutralized by the addition of 5 mL 0.1 M HCl and diluted to 25 mL with diluent.

To prepare the blank, 5 mL of 0.1 M NaOH and 5 mL of 0.1 M HCl were diluted to 25 mL with diluent.

#### 3.5.3. Hydrolytic Degradation

Twenty-five milligram micafungin sodium were accurately weighed and dissolved in 5 mL of diluent, 10 mL of water were added and the mixture was kept at 70 °C for 1 h. The solution were brought to room temperature and diluted to 25 mL with diluent.

To prepare the blank, 10 mL of water were diluted to 25 mL with diluent.

#### 3.5.4. Oxidative Degradation

Twenty-five milligram micafungin sodium were accurately weighed and dissolved in 5 mL of diluent, 5 mL of 3% hydrogen peroxide were added and the mixture were kept at room temperature for 1 h. The solution was diluted to 25 mL with diluent.

To prepare the blank, 5 mL of 3% hydrogen peroxide was diluted to 25 mL with diluent.

#### 3.5.5. Thermal Degradation

Thermal degradation was performed by spreading the drug substance in a petri dish as a thin film at 105 °C for 24 h. Twenty-five milligram of thermal degradation sample were accurately weighed, dissolved and diluted to 25 mL with diluent.

#### 3.5.6. Photolytic Degradation

Photodegradation was performed by spreading the drug substance in a petri dish as a thin film and keeping it in a photostability chamber equipped with ultraviolet light with overall illumination of >1.2 million lux hours with an energy of not less than 200 Wh/m^2^. Twenty-five milligram of photolytic degradation sample were accurately weighed, dissolved and diluted to 25 mL with diluent.

## 4. Conclusions

In this paper, a sensitive, specific, accurate, validated and well-defined stability-indicating HPLC method for the determination of micafungin sodium in the presence of degradation products and its related substances was described. The behavior of micafungin sodium under various stress conditions was studied and the results have been presented. All the degradation products formed during application of stress conditions, and process impurities, were well separated from the drug substance, indicating the method was stability-indicating. The information presented here could be very useful for monitoring the quality of bulk drug and could be used to check the quality of the drug during stability studies.

## Figures and Tables

**Table 1:** System suitability parameters.

Analyte	*R*	*N*	Symmetry factor	RRT	*t*_R_ ± SD (min)	RSD (%)
1	/	14010	1.08	0.44	14.50 ± 0.05	0.34
2	13.11	14700	1.12	0.69	23.12 ± 0.07	0.30
3	7.55	15588	1.22	0.85	28.63 ± 0.08	0.27
4	1.65	17556	1.24	0.91	30.34 ± 0.07	0.23
a M	3.18	18109	1.09	1.00	33.51 ± 0.07	0.21
5	3.22	18299	1.11	1.10	36.88 ± 0.07	0.19
6	4.87	17666	1.22	1.27	42.77 ± 0.08	0.17

a micafungin sodium;

*N*, theoretical plates; *R*, resolution; RRT, relative retention time; *t*_R_, retention time and RSD relative; standard deviation obtained from five replicate injections.

**Table 2:** Precision-repeatability and precision-intermediate of impurities of micafungin sodium.

Analyst	% RSD impurities 1–6					

	Imp-1	Imp-2	Imp-3	Imp-4	Imp-5	Imp-6
Analyst 1 (*n* = 6)	0.22	0.17	0.27	0.18	0.16	0.18
Analyst 2 (*n* = 6)	0.33	0.26	0.34	0.26	0.24	0.25
Analyst 1 and 2 (*n* =12)	0.36	0.55	0.28	0.38	0.37	0.35

Repeatability for a solution of micafungin sodium (1000 μg/mL) spiked with 0.5% of each available impurity.

**Table 3:** Limit of detection (LOD), limit of quantification (LOQ) and regression.

Analyte	LOD (μg/mL)	LOQ (μg/mL)	Repeatability at LOQ (% RSD, *n* = 6)	Linearity range (μg/mL)	Calibration equation (*y* = area, *x* = μg/mL)	95% confidence interval for intercept	Residual standard deviation
Imp-1	0.06	0.19	1.36	0.19–7.50	*y* = 23.382*x* − 1.3075	(−3.81,1.20)	1.28
Imp-2	0.11	0.26	1.55	0.26–7.50	*y* = 25.474*x* + 0.136	(−1.20,1.47)	0.62
Imp-3	0.12	0.29	1.28	0.29–7.50	*y* = 25.664*x* − 2.9776	(−3.81,2.60)	1.61
Imp-4	0.12	0.31	1.83	0.31–7.50	*y* = 21.13*x* + 0.4211	(−1.20,2.03)	0.81
Imp-5	0.08	0.25	1.15	0.25–7.50	*y* = 20.019*x* − 0.5906	(−2.67,1.49)	1.05
Imp-6	0.13	0.35	1.66	0.31–7.50	*y* = 20.455*x* − 0.6923	(−2.63,1.26)	0.97
a M	0.09	0.21	1.27	0.21–7.50	*y* = 21.049*x* + 0.4519	(−1.07,1.97)	0.79

a micafungin sodium.

**Table 4:** The percent recovery of related substances of micafungin sodium from triplicate preparations.

Analyte	Addd (μg/mL)	Measured mean ± SD (μg/mL)	Recovery (%)
1	2.50	2.48 ± 0.012	99.2
	5.00	4.92 ± 0.039	98.4
	7.50	7.63 ± 0.072	101.7

2	2.50	2.51 ± 0.026	100.4
	5.00	5.03 ± 0.037	100.6
	7.50	7.59 ± 0.108	101.2

3	2.50	2.55 ± 0.028	102.0
	5.00	4.95 ± 0.066	99.0
	7.50	7.39 ± 0.113	98.5

4	2.50	2.52 ± 0.023	100.8
	5.00	5.08 ± 0.056	101.6
	7.50	7.61 ± 0.087	101.5

5	2.50	2.53 ± 0.019	101.2
	5.00	4.91 ± 0.036	98.2
	7.50	7.62 ± 0.106	101.6

6	2.50	2.48 ± 0.021	99.2
	5.00	4.98 ± 0.046	99.6
	7.50	7.64 ± 0.107	101.9

**Table 5:** Robustness data.

Type of change	Variation	Retention time of principal (min)	Resolution					

			1/2	2/3	3/4	4/a M	a M/5	5/6
flow rate (mL/min)	0.9	37.17	13.67	7.50	1.64	3.21	3.21	4.88
	1.0	33.51	13.32	7.45	1.65	3.15	3.14	4.82
	1.1	30.58	13.06	6.95	1.54	3.08	3.07	4.59

Temperature (°C)	40	34.79	13.11	7.18	1.42	3.08	3.28	5.19
	45	33.51	13.32	7.45	1.65	3.15	3.14	4.82
	50	32.74	13.61	7.8	1.79	3.14	3.11	4.48

Acetonitrile ratio (%)	37	42.76	14.53	7.41	1.46	2.8	3.27	4.74
	38	33.51	13.32	7.45	1.65	3.15	3.14	4.82
	39	28.53	12.96	7.35	1.28	2.85	3.01	4.58

pH	2.7	36.05	13.84	7.56	1.55	3.19	3.15	4.61
	2.9	33.51	13.32	7.45	1.65	3.15	3.14	4.82
	3.1	37.49	13.82	7.73	1.47	3.23	3.2	4.61

a micafungin sodium.

**Table 6:** Summary of forced degradation results.

Stress condition/media/duration	Degradation (%)	Number of impurities	Retention time (*t*_R_) (min)	Peak purity
Acidic/0.1M HCl/RT/1 h	11.1	5	9.43; 12.25; 17.32; 23.13; 42.44	0.99965
Alkaline/0.1M NaOH/RT/1 h	25.1	9	7.72; 8.82; 12.21; 17.12; 19.08; 23.13; 27.35; 28.11; 42.44	0.99972
Neutral/H_2_O/70 °C/1 h	5.5	5	12.18; 14.14; 17.18; 23.13; 42.44	0.99981
Oxidative/3% H_2_O_2_/1 h	17.6	4	6.53; 12.25; 18.32; 23.13	0.99989
Photolytic/UV-lamp/72 h	9.2	3	13.67; 18.29; 23.13	0.99977
Thermal/105 °C/24 h	5.2	3	13.67; 18.29; 23.13	0.99998

**Table 7:** Analysis of related substances of micafungin sodium in bulk samples by high performance liquid chromatography (HPLC).

Analyte	Impurities%							

	1	2	3	4	5	6	Any unknown Impurity (%)	Total impurities (%)
1228065	<LOD	0.19	0.068	<LOD	0.057	<LOD	ND	0.315
1228066	<LOD	0.19	<LOD	<LOD	0.068	<LOD	ND	0.258
1228067	0.11	0.16	<LOD	<LOD	0.059	<LOD	ND	0.219

ND, Not detected.
